# Supplementary material for: Splice-Junction-Based Mapping of Alternative Isoforms in the Human Proteome
Source: Cell Rep. Author manuscript; Available in PMC 2020 Jan 15. (PMC6961840; doi:10.1016/j.celrep.2019.11.026)
Supplement: 3 [file NIHMS1546469-supplement-3.zip › DF2/PXD0010154/Liver-267-Q14244-WSWGGSLHGSPSIHSAAR.pdf]

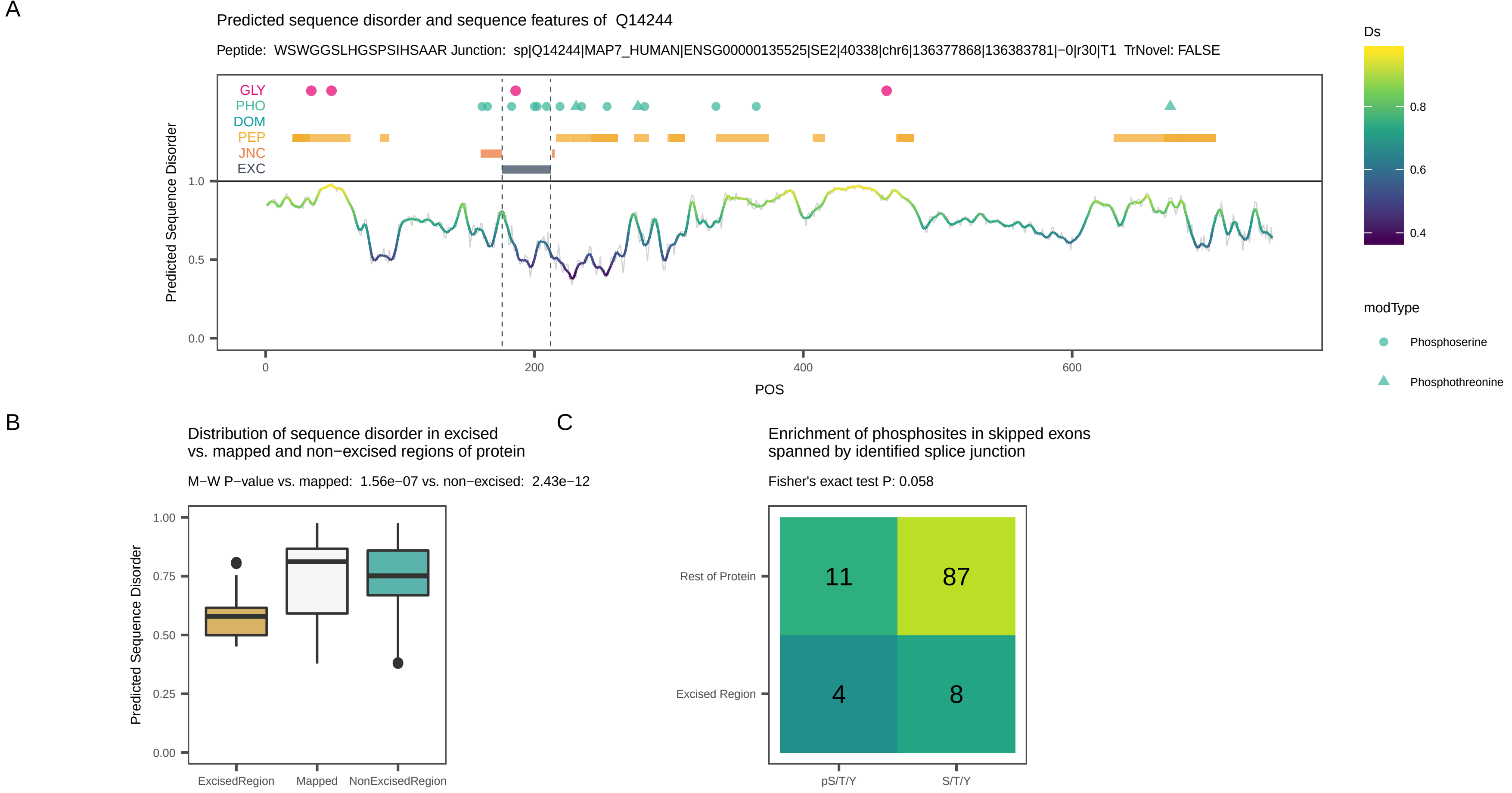
A
Predicted sequence disorder and sequence features of Q14244
Peptide: WSWGGSLHGSPSIHSAAR Junction: sp|Q14244|MAP7_HUMAN|ENSG00000135525|SE2|40338|chr6|136377868|136383781|−0|r30|T1 TrNovel: FALSE
GLY
PHO
DOM
PEP
JNC
EXC
Predicted Sequence Disorder
1.0
0.5
0.0
0
200
400
600
POS
Ds
0.8
0.6
0.4
modType
Phosphoserine
Phosphothreonine
B
Distribution of sequence disorder in excised
vs. mapped and non−excised regions of protein
M−W P−value vs. mapped: 1.56e−07 vs. non−excised: 2.43e−12
Predicted Sequence Disorder
1.00
0.75
0.50
0.25
0.00
ExcisedRegion
Mapped
NonExcisedRegion
C
Enrichment of phosphosites in skipped exons
spanned by identified splice junction
Fisher's exact test P: 0.058
Rest of Protein
Excised Region
11
87
4
8
pS/T/Y
S/T/Y
